# Do Motor Imagery Performances Depend on the Side of the Lesion at the Acute Stage of Stroke?

**DOI:** 10.3389/fnhum.2016.00321

**Published:** 2016-06-27

**Authors:** Claire Kemlin, Eric Moulton, Yves Samson, Charlotte Rosso

**Affiliations:** ^1^APHP, Urgences Cérébro-Vasculaires, Hôpital Pitié-SalpêtrièreParis, France; ^2^APHP, Service de Médecine Physique et Réadaptation, Hôpital Pitié-SalpêtrièreParis, France; ^3^Centre de Recherche de l’Institut du Cerveau et de la Moelle épinièreParis, France; ^4^UPMC Paris 6, INSERM, U1127; CNRS, UMR 7225Paris, France; ^5^CONAM, UPMC Paris 6, INSERM, U1127, CNRS, UMR 7225Paris, France; ^6^COGIMAGE, UPMC Paris 6, INSERM, U1127, CNRS, UMR 7225Paris, France

**Keywords:** stroke, motor imagery, mental practice, recovery

## Abstract

Motor imagery has been considered a substitute for overt motor execution to study post-stroke motor recovery. However, motor imagery abilities at the acute stage (<3 weeks) are poorly known. The aim of this study was to compare explicit and implicit motor imagery abilities in stroke patients and healthy subjects, correlate them with motor function, and investigate the role of right or left hemisphere lesions on performance. Twenty-four stroke patients at the acute stage and 24 age- and gender-matched healthy volunteers performed implicit (Hand Laterality Judgment Task) and explicit (number of imagined/executed hand movements) motor imagery tasks and a clinical motor assessment. Differences between healthy subjects and patients as well as the impact of lesion side on motor imagery were studied using ANOVA. We analyzed the relationship between motor executed and imagined movements (temporal congruence) using Pearson correlations. Our study shows that for implicit imagery, stroke patients had slower reaction times [RTs, *t*(46) = 1.7, *p* = 0.02] and higher error rates for the affected hand [*t*(46) = 3.7, p < 0.01] yet shared similar characteristics [angle effect: *F*(1,46) = 30.8, *p* ≤ 0.0001] with respect to healthy subjects. For the unaffected hand, right-sided stroke patients had a higher error rate and similar RTs whereas left sided stroke had higher RTs but similar error rate than healthy subjects. For explicit imagery, patients exhibited bilateral deficits compared to healthy subjects in the executed and imagined condition (*p* < 0.0001). Patients and healthy subjects exhibited a temporal congruence between executed and imagined movements (*p* ≤ 0.04) except for right-sided strokes who had no correlation for both hands. When using motor imagery as a tool for upper limb rehabilitation early after stroke, caution must be taken related to the side of the lesion.

## Introduction

Motor imagery shares a number of similarities with overt movement execution such as behavioral (Jeannerod, 1995), physiological parameters (Kranczioch et al., 2009), and perhaps more importantly, certain functional neuroanatomical correlates (e.g., recruitment of brain motor networks; Confalonieri et al., 2012). Motor imagery has been used in upper limb rehabilitation to improve post-stroke motor function (Page et al., 2011), pain (Moseley, 2006), neglect (Welfringer et al., 2011), or daily living activities (Liu et al., 2004), mostly at the subacute and the chronic stage. Two randomized controlled trials have trained patients by mental practice at the acute stage (Liu et al., 2004; Rosa et al., 2010) but one examined specifically motor function in a small sample of patients (*n* = 17). Yet, a clear understanding of whether and how mental simulation performance is modified by motor stroke and when is needed, especially at the acute stage. Motor imagery is actually an umbrella term that includes two different types: implicit and explicit mental imagery (Di Rienzo et al., 2014). Implicit motor imagery concerns the ability to perform mental rotation, usually with one part of the body, by a first person perspective. It can be tested by the Hand Laterality Judgment Task (HLJT) in which a subject has to determine the laterality (handedness) of pictures of hands (De Vries et al., 2011; Yan et al., 2013). In this type of task, stroke patients are susceptible to exhibit decreases in accuracy, RTs, or both. Explicit imagery is the internal rehearsal of a movement (for example, a fist closure task) that could be imagined visually or kinesthetically (Malouin et al., 2012; Wong et al., 2013). In the context of explicit imagery, the number of executed and imagined movements in a given amount of time (temporal congruence) has also been suggested to be altered in stroke patients (Di Rienzo et al., 2014). It is, however, worth noting that studies investigating these performances have been performed at the chronic phase (>3 months) except in one study (De Vries et al., 2011). In other words, motor imagery abilities at the acute stage of stroke are not well known and may be of importance to use mental practice as a tool in upper limb rehabilitation, as soon as possible. In addition, the impact of the side of the lesion on motor imagery has been questioned in a recent review (Di Rienzo et al., 2014), and for both types of motor imagery. In order to better characterize the abilities of stroke patients in implicit and explicit motor imagery, we performed a behavioral study in acute stroke patients and healthy individuals. First, we compared the characteristics of implicit and explicit imagery in healthy subjects and stroke patients. We then investigated which type of motor imagery was impaired specifically in right- and left-sided lesions. Finally, we correlated the motor imagery performance with motor function in patients.

## Materials and Methods

### Participants

Twenty-four stroke patients and 24 age- [*t*(46) = 0.47; *p* = 0.64] and gender-matched (*p* = 0.78) healthy volunteers were recruited between February 2014 and September 2015. Characteristics of patients and healthy subjects are given in **Table 1**. Patients were recruited from the stroke unit at the Hôpital La Pitié-Salpêtrière. Inclusion criteria were a cerebrovascular accident <3 weeks, proven by MRI and a score >0 for the upper limb motor item on the NIHSS (National Institute of Health Stroke Scale). Exclusion criteria were severe cognitive dysfunction (MMSE < 24), severe aphasia (inability to understand test instructions), and visual problems including hemianopia that could interfere with this study. Healthy individuals with no history of neurological or psychiatric disease were recruited. Motor imagery capacities of the subjects were tested with a self-assessment questionnaire inspired by the Kinesthetic and Visual Imagery Questionnaire (KVIQ). Two domains (visual and kinesthetic) constituted the questionnaire (Malouin et al., 2007) which entailed imagining a movement of opening and closing one’s hand. Participants had to rate the vividness of clarity of the image (visual motor imagery) and intensity of sensation (kinesthetic motor imagery) on a five-point ordinal scale from *1 = no image/no sensation* to *5 = image/sensation as if the movement was seen/executed*. The institutional review board (IRB) of Paris VI University approved the study, and consent was obtained from each participant.

**Table 1:** Characteristics of Patients and Healthy Subjects.

	Patients	Healthy subjects
Age (years)	64.9 ± 13.6	63.2 ± 14.8
Gender (% of Males)–n	54% –13	54% –13
JTT affected hand (seconds) ^∗^	180.5 ± 174.7	31.2 ± 4.3
JTT unaffected hand (seconds) ^∗^	45.9 ± 12.1	30.9 ± 4.7
JTT ratio ^∗^	4.1 ± 4.0	1.0 ± 0.1
Pinch Grip affected hand (Newtons) ^∗^	104 ± 64	264 ± 93
Pinch Grip unaffected hand (Newtons) ^∗^	186 ± 77	277 ± 90
Pinch Grip ratio ^∗^	0.6 ± 0.2	1.0 ± 0.1
Kinesthetic score (KVIQ) affected hand	2.4 ± 1.3	2.6 ± 1.3
Kinesthetic score (KVIQ) unaffected hand	2.8 ± 1.4	2.7 ± 1.4
Visual score (KVIQ) affected hand	3.0 ± 1.1	3.1 ± 1.4
Visual score (KVIQ) unaffected hand	3.4 ± 1.1	3.0 ± 1.3

*Results are presented as mean ± SD.*

*JTT, Jebsen Taylor Hand Function Test; KVIQ, Kinesthetic and Visual Imagery Questionnaire, ^∗^p < 0.05.*

### Testing Procedure

The testing procedure was similar for all participants and done by the same physiotherapist.

#### Motor Ability Assessment

Patients motor ability was evaluated within 3-weeks post-stroke onset with two scales. The first was the Jebsen–Taylor Hand Function Test (JTT) (Jebsen et al., 1969). As Hummel et al. (2005) we included only six of the seven JTT subtests: turning over cards, picking up small objects, picking up beans with a teaspoon, stacking checkers, moving large light cans, and moving heavy cans. Each subtest was timed, and the total JTT time was computed by adding each sub-item duration. JTT ratio was computed by dividing the JTT time of the paretic hand by that of the unaffected hand. The second evaluation served to measure force grip strength using a dynamometer (MIE, Medical Research Ltd.,)^1^. The maximal handgrip strength (mGS) was recorded three times then averaged. The mGS ratio was calculated as the mGS of the affected hand divided by that of the unaffected hand (Rosso et al., 2013).

#### Implicit Motor Imagery Assessment

**FIGURE 1:**
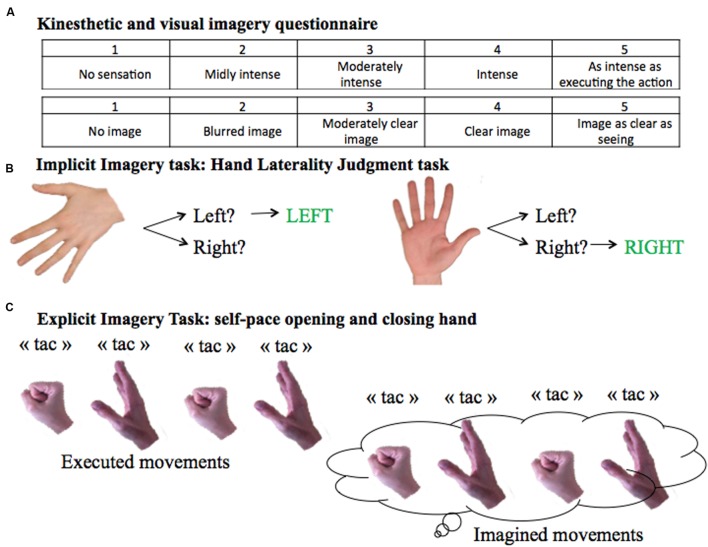
**Motor Imagery Tasks. (A)** KVIQ scoring system from 1 to 5 for the visual and the kinesthetic scores. **(B)** Implicit imagery task: Hand Laterality Judgment Task. **(C)** Explicit imagery task: self-pace hand opening and closing.

Patients completed a computerized Hand Laterality Judgment Task (HLJT; Parsons, 1987; **Figure 1**). The task consisted of displaying on a computer screen the palm and backside of the left and the right hands from a single participant (De Vries et al., 2013). The pictures of the hands were presented at six different angles (0°, 60°, 120°, 180°, 240°, and 300°). The orientation of the hand pointing upward was defined as an angle of 0°. The orientations 0°, 60°, and 300° were defined to be anatomical angles whereas 120°, 180°, and 240° were defined to be non-anatomical angles. There were therefore 24 unique images, presented three times, resulting in 72 total images. Pictures were presented in a computer-made random order using E-Prime 2.0 (Psychology Software Tools, Inc, Pittsburgh, PA, USA), and patients were asked to identify as fast and accurately as possible whether the picture showed a left or right hand. Before the start of the test, participants practiced the task until they felt comfortable with the computer environment and the mouse system, in order to avoid biases due to learning. For each picture, the average RT (i.e., the time between onset of image display and button press) for correct responses and the error rate (number of incorrect responses over the total number of images) were calculated.

#### Explicit Motor Imagery Assessment

To evaluate explicit motor imagery ability, subjects were asked to open and close one hand at a time at their own pace (**Figure 1**). The test was performed twice: first by overtly executing, then by imagining the movement. In order to count the number of imagined movements, subjects were instructed to verbally signal each time they opened and closed their hand by saying “tac”. Patients were told to stop after 15 s. The task was performed with both hands in a counterbalanced order.

### Statistical Analysis

The descriptive statistics are presented as mean ± SD. All statistical analyses were performed using SPSS software (version 20). The statistical level of significance was set at *p* < 0.05.

#### Comparison of Implicit and Explicit Motor Imagery Abilities between Healthy Subjects and Stroke Patients

In order to control for the effects of handedness in comparing patients performance with those of healthy controls, we employed a method previously reported (Nowak et al., 2014). The method consists of pairing each patient with a unique control subject matched in age, gender, and handedness, regardless of the affected hand. For example, if the paretic hand of a right-handed patient was the left hand, it would then be matched with the left hand of a right-handed healthy subject. We refer to this hand in healthy subjects as the paired-affected hand. Consequently, the right hand of the same healthy subject is referred to as the paired-unaffected hand. To describe and compare the characteristics of implicit and explicit imagery in healthy subjects and acute stroke patients, we performed repeated-measures ANOVA on behavioral data with a between-subject factor GROUP and several within-subject factors. First, for the HLJT task, the behavioral data were RTs and error rates; the within-subject factors were HAND (affected or paired-affected-AFF vs. non-affected or paired-unaffected-UNAF), ANGLE (anatomical vs. non-anatomical orientations), and POSITION (palm vs. backside views). For the explicit motor imagery task, the behavioral data consisted of the number of movements during a 15-s period with two within-subject factors: HAND (AFF and UNAF) and CONDITION (Executed-EM vs. imagined-IM). Following the ANOVAs, *t*-tests were performed. The relationship between imagination and execution times (temporal congruence) was also studied with Pearson correlations.

#### Impact of the Side of the Lesion on Motor Imagery Performances

The same ANOVAs were carried out for implicit and explicit task scores with the between-subject factor SIDE instead of GROUP (right- vs. left-sided strokes). For the explicit task analysis, temporal congruence was tested with the Pearson correlations in each group (right- vs. left-sided strokes).

#### Correlation between Motor Imagery and Clinical Outcome

Due to the non-normal distribution of clinical scores, Spearman correlations between the three motor scores (JTT times and ratio, mGS ratio) and implicit (error rates, RTs) and explicit (number of movements) motor imagery performance were computed.

## Results

Seventeen (71%) patients suffered from an ischemic stroke (7 from atherosclerosis, 3 from cardioembolic sources, 3 cryptogenic, 2 small vessel disease, 1 vasculitis, and 1 cervical artery dissection) and seven (29%) from an intra-cerebral hemorrhage (4 from hypertension, 1 from anti-vitamin K agent and 2 unknown causes). Fourteen (58%) patients were subcortical strokes, 9 (38%) cortical and 1 (4%) was cortico-subcortical strokes. Patients (mean ± SD delay since stroke: 9 ± 5 days) had lower grip strength and less dexterity (JTT) in the affected than the unaffected hand and than healthy subjects in both hands (*p* < 0.001 for all measures; **Table 1**). The mean ± SD upper limb motor item of the NIHSS was 1.0 ± 1.2. Patients exhibited similar results compared to healthy subjects for the KVIQ in both visual and kinesthetic scores (*p* > 0.05 for all items). Out of 24 patients, 12 (50%) had a right-sided stroke. Right-sided stroke patients had similar motor scores for the NIHSS, JTT, and mGS than left-sided stroke patients (**Table 2**).

**Table 2:** Characteristics of left-sided and right-sided stroke patients.

Mean, SD	Left-sided stroke patients	Right-sided stroke patients
Age (years)	67.3 ± 14.7	62.6 ± 12.6
Gender (% of Males)–n	50–6	58 -7
JTT affected (seconds)	211.9 ± 221	145.7 ± 103
JTT unaffected (seconds)	45.6 ± 10.6	46.2 ± 14.1
JTT ratio	5.0 ± 5.2	3.1 ± 1.8
Pinch Grip affected (Newtons)	95 ± 43	115 ± 81
Pinch Grip unaffected (Newtons)	183 ± 88	189 ± 67
Pinch Grip ratio	0.6 ± 0.2	0.6 ± 0.3
Delay since stroke (days)	9 ± 5	9 ± 6
RTs (affected hand) (seconds)	6.1 ± 5.6	3.5 ± 1.0
RTs (unaffected hand) (seconds)^†^	6.4 ± 6.7	2.7 ± 0.8
Error rates (affected hand) (%)	15 ± 9	13 ± 9
Error rates (unaffected hand) (%)^∗^	12 ± 7	16 ± 10
Number of movements EM (affected hand)	10 ± 3	9 ± 2
Number of movements IM (affected hand)	8 ± 4	7 ± 3
Number of movements EM (unaffected hand)	11 ± 4	12 ± 4
Number of movements IM (unaffected hand)	9 ± 4	8 ± 2

*^∗^p < 0.05; ^†^p < 0.1. JTT, Jebsen Taylor Hand Function Test.*

### Implicit Motor Imagery Abilities

Descriptive statistics of RTs and error rates are given in **Table 3**.

**Table 3:** Performance of stroke patients and healthy subjects in the HLJT.

	Reaction times (s)		Error rates (%)	
	Patients sec	Healthy Subjects sec	Patients %	Healthy Subjects %
Total	4.5 ± 4.7†	2.9 ± 1.5	32 ± 14^∗^	19 ± 9
Anatomical orientation	4.3 ± 4.8†	2.4 ± 0.9	13 ± 9^∗^	7 ± 5
Non-anatomical orientation	5.4 ± 5.3†	3.3 ± 1.6	16 ± 8^∗^	11 ± 5
Palmside position	5.2 ± 5.3^∗^	2.9 ± 1.4	14 ± 8^∗^	8 ± 6
Backside position	4.3 ± 4.3†	2.8 ± 1.1	15 ± 10^∗^	10 ± 7
Affected (paired-affected) hand	4.8 ± 4.1^∗^	2.8 ± 1.2	16 ± 9^∗^	9 ± 5
Unaffected (paired-unaffected) hand	4.5 ± 4.8†	2.8 ± 1.2	16 ± 8^∗^	10 ± 5

*^∗^p < 0.05, ^†^p < 0.1. Superscripts adjacent to patient statistics indicate significant differences from healthy controls.*

**FIGURE 2:**
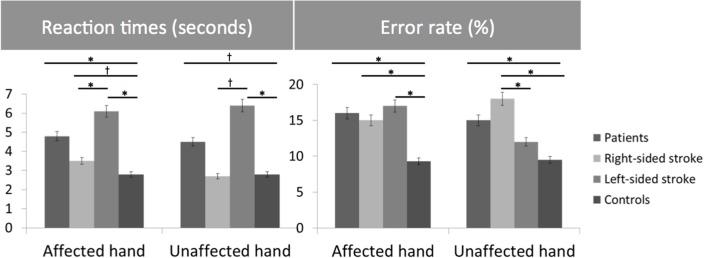
**Bar graphs of the reaction times (RTs) (seconds) and error rates (%) for the Hand Laterality Judgment Task in patients, healthy controls, right- and left-sided stroke patients for the affected and unaffected hand.** Height of bars is given as the mean and error bars are given as the 5% standard error. ^∗^*p* < 0.05, ^†^*p* < 0.1.

Healthy subjects and patients had longer RTs for non-anatomical than anatomical angles [ANGLE effect: *F*(1,46) = 30.8, *p* < 0.0001] and for palm- than backside views [POSITION effect: *F*(1,46) = 5.5, *p* = 0.02]. *Post hoc t*-tests demonstrated that the POSITION effect was driven by the patients’ group [and not the healthy subjects, *t*(23) = –0.86, *p* = 0.39]. Compared to healthy subjects, patients had longer RTs in recognizing the affected hand pictures [*t*(46) = 1.7, *p* = 0.02] with a non-significant similar trend for the unaffected hand [*t*(46) = 2.3, *p* = 0.10] (**Figure 2**). When considering the side of the lesion, left-sided stroke patients were slower than right-sided stroke and healthy subjects in recognizing both pictures of the affected [*t*(22) = 1.9, *p* < 0.001 and *t*(22) = 2.8, *p* = 0.008] and the unaffected hands pictures [*t*(22) = 1.6, *p* = 0.05 and *t*(22) = 2.6, *p* = 0.01]. In contrast, right-sided patients had preserved abilities compared to healthy subjects for the unaffected hand pictures [*t*(34) = –0.3, *p* = 0.73] and tended to be slower for the affected hand pictures [*t*(34) = 1.8, *p* = 0.08].

For the error rate, patients made more errors than healthy subjects [*t*(46) = 3.7, *p* = 0.0007]. Healthy subjects and patients had higher error rates for non-anatomical than anatomical angles [ANGLE effect: *F*(1,46) = 21.2, *p* < 0.0001]. No effect of POSITION [*F*(1,46) = 1.2, *p* = 0.27] was found. The side of the lesion influences differently the error rate depending on the handedness of the pictures [HAND^∗^SIDE interaction: *F*(1,22) = 6.9, *p* = 0.01]. Indeed, right-sided strokes had higher error rates for the affected hand and tended to have for the unaffected hand pictures than left-sided strokes [*F*(1,22) = 4.2, *p* = 0.008 and *F*(1,22) = 0.3, *p* = 0.05] and healthy subjects [*t*(34) = –4.1, *p* = 0.001, and *t*(34) = 2.8, *p* = 0.003]. In contrast, left-sided strokes had similar error rates than healthy subjects for the unaffected hand pictures [*t*(34) = 1.3, *p* = 0.26], and made more errors for the affected hand pictures [*t*(34) = 3.4, *p* = 0.008].

### Explicit Motor Imagery Abilities

**FIGURE 3:**
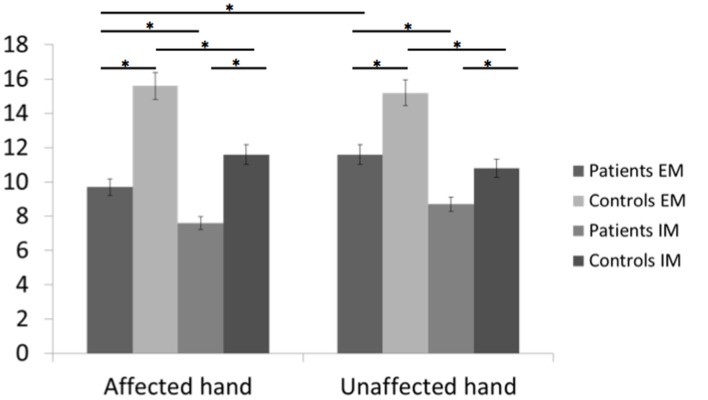
**Bar graphs of the number of executed and imagined movements for the affected and unaffected hand in patients and healthy controls.** Height of bars is given as the mean and error bars are given as the 5% standard error. ^∗^*p* < 0.05.

Descriptive statistics of the number of movements in the two conditions are given in **Table 4** (**Figure 3**).

**Table 4:** Performance in the explicit motor imagery task for stroke patients and healthy subjects.

	Number of movements (EM)		Number of movements (IM)	
	Patients	Healthy subjects	Patients	Healthy subjects
Affected (Paired-AFF) Hand	10 ± 3^∗^	16 ± 7	8 ± 3^∗^	12 ± 5
Unaffected (Paired-UNAF) Hand	12 ± 4^∗^	15 ± 8	9 ± 3^∗^	11 ± 4

*^∗^p < 0.05. Superscripts adjacent to patient statistics indicate significant differences from healthy controls. IM, imagined movements; EM, executed movements.*

In healthy subjects and patients, the number of executed movements was higher than the number of imagined movements for both hands (CONDITION effect [*F*(1,46) = 31.8, *p* < 0.0001]. There was also a HAND^∗^GROUP interaction [*F*(1,46) = 14.8, *p* < 0.0001] and a HAND^∗^CONDITION interaction [*F*(1,46) = 4.4, *p* = 0.04]. In other words, patients had a smaller number of movements for both hands than healthy subjects [EM: *t*(46) = –3.5, *p* = 0.0001 and *t*(46) = –2.1, *p* = 0.045 for the affected/unaffected hand, IM *t*(46) = –3.4, *p* = 0.001, and *t*(46) = –2.07, *p* = 0.03 for the affected/unaffected hand]. Moreover, the comparison between the affected vs. the unaffected hand revealed no significant differences for the healthy subjects in the IM [*t*(23) = –1.8, *p* = 0.57] and the EM conditions [*t*(23) = –1.0, *p* = 0.83]. In contrast, as expected patients had a smaller amount of executed movement of the affected vs. the unaffected hand [*t*(22) = 3.3, *p* = 0.005], reflecting the neurological deficit. But, the number of movements over a 15-s period was similar for the unaffected hand than for the affected hand for the IM condition [*t*(22) = 1.9, *p* = 0.21].

For the comparison between right-and left-sided stroke patients, there was a significant triple interaction HAND^∗^CONDITION^∗^SIDE [*F*(1,22) = 4.6, *p* = 0.04]. First, as expected, for the affected hand, the number of executed movements was smaller than the one of healthy subject [*t*(32) = –2.7, *p* = 0.01 for right- and *t*(34) = –2.5, *p* = 0.01 for left-sided stroke patients]. The number of movements in the imagined condition was also smaller for the affected hand [*t*(34) = –3.1, *p* = 0.004 for right- and *t*(34) = –2.1, *p* = 0.03 for left-sided stroke patients]. For the unaffected hand, there were no significant differences except for the smaller number of imagined movements for right-sided strokes comparing to healthy subjects [*t*(34) = –3.1, *p* = 0.03].

**FIGURE 4:**
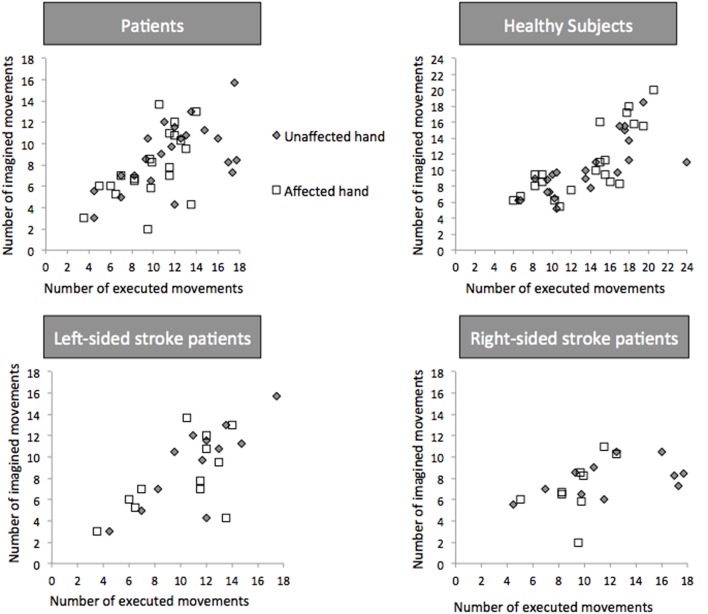
**Temporal Congruence for Explicit Motor Imagery.** Number of executed and imagined movements in patients, healthy controls, left- and right-sided patients. White square dots and gray diamonds represent movements for the affected and unaffected hand, respectively.

Temporal congruence [i.e., the correlation between the number of movements in the executed and imagined conditions (**Figure 4**)] was present for both hands in healthy subjects (*r* = 0.723, 95% CI: 0.553-0.887, *p* < 0.0001 for the paired-affected hand and 0.797, 95% CI: 0.696–0.928, *p* < 0.001 for the paired-unaffected hand) and stroke patients (*r* = 0.525, 95% CI: 0.320–0.757, *p* = 0.08 for the affected hand and *r* = 0.506, 95% CI: 0.252–0.785, *p* = 0.01 for the unaffected hand).

In left-sided patients (**Figure 4**), temporal congruence was present for the unaffected hand (*r* = 0.810, 95% CI: 0.226–0.964; *p* = 0.001) and the affected hand (*r* = 0.595, 95% CI: 0.033–0.817; *p* = 0.04). In right-sided strokes temporal congruence was not present for both hands (*p* = 0.27 and 0.25).

### Correlation between MI Performance and Clinical Motor Scores

No correlation was found between either implicit (RTs, error rates) or explicit task performance (number of movements in the imagined condition) with clinical motor scores (the JTT times or mGS ratios).

## Discussion

We have confirmed that stroke patients have impaired motor imagery abilities at the acute stage. Second, the effect of the side of the stroke was not the same for the implicit and the explicit motor imagery tasks.

### Implicit Imagery Tasks Impairment in Stroke and Impact of Lesion Side

**FIGURE 5:**
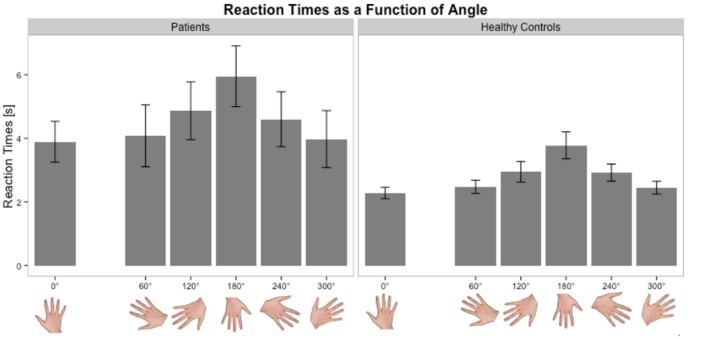
**Distribution of RTs as a function of angle in the Hand Laterality Judgment Task in patients and healthy controls.** Height of bars is given as the mean and error bars are given as the standard error of the mean.

Stroke patients at the acute stage had slower RTs and higher error rates than healthy subjects for the affected hand. The error rate (32%) was higher in our population than in a previous study (14%) (Yan et al., 2013). This discrepancy could be explained not only by the older age of our population (mean = 67 vs. 60 years in Yan et al., 2013) but also by the fact that the study was conducted early after stroke. Several studies have shown a selective decline in performance with age in healthy subjects, most likely related to a decline in visuospatial and kinesthetic abilities (Skoura et al., 2008; Malouin et al., 2010). For the unaffected hand, the results depended on the side of the lesion. Left- and right-sided stroke patients experienced difficulties in performing the task. But, the way to solve it was different. For right-sided lesions, RTs were similar but the error rate was higher than healthy subjects. In contrast, left sided strokes exhibited an inverse profile, meaning that the error rate was similar but they took more time than healthy subjects to answer. This was consistent with a previous study in 11 left-sided stroke patients who presented comparable and slow RTs between right and left hands (Yan et al., 2013). Therefore, implicit mental imagery is a complex task involving cognitive and motor processes (Lehéricy et al., 2004). It requires solving problems within working memory, body schemas, visuospatial information, motor planning, motor control, and decision making, each of these could specifically be impaired in stroke patients. Some characteristics of implicit imagery were preserved in our patients, such as the angle effect. In both groups, anatomical orientations were easier to recognize than non-anatomical orientations. Moreover, Fitts’s law (increase in RTs as angle deviates from 0°) was verified in both groups (**Figure 5**). The fact that the ANGLE effect was found significant in stroke patients for both RTs and accuracy is in favor of at least a partial respect of body schema representation or visuospatial processing in our sample. Actually, the inverse profile of left-sided and right-sided stroke could reflect a difference in motor decision-making process (Behan et al., 2015). There are growing evidences regarding the role of the right inferior frontal gyrus during a particular form of executive control referred to as response inhibition, i.e., the ability to suppress irrelevant responses to a stimulus. In case of right hemisphere lesions, this fronto-parietal network is disturbed and impulsive responses (explaining the high error rates with preserved RTs) occur (Garavan et al., 1999). Cojan et al. (2009) also demonstrated that in motor inhibition, right frontal areas were activated by nogo trials in healthy subjects. Moreover, they found that there was no hemispheric lateralization for motor inhibition in the normal state. For left-sided stroke, the right hemisphere is functional and impulsive responses are inhibited (similar error rate than healthy subjects).

### Explicit Imagery Task Impairment in Stroke and Impact on Lesion Side

As expected, patients were faster in a self-paced condition for executed movements of the unaffected than the affected hand, reflecting the motor impairment of the upper limb. In patients, the number of imagined movements was similar for the unaffected than the affected hand. Nevertheless, patients were slower at imagining movements for both hands than healthy subjects. Another important result is that the number of imagined movements was smaller than that of executed movements for both hands (Sabaté et al., 2004; Stinear et al., 2007). Possible explanations could be either the lack of sensorimotor feedback that creates extra difficulty for stroke patients. Proprioceptive feedback has been shown to bridge cortical networks and abilities between motor imagery and execution (Bauer et al., 2015), and to induce behavioral gains in stroke patients (Brauchle et al., 2015; Naros and Gharabaghi, 2015). Alternatively, another possibility to explain the discrepancy between frequencies of executed and imagined movements could be the increased mental effort in performing internal rehearsal as already described in post-stroke dystonic patients (Lehéricy et al., 2004). Sabaté et al. (2004) in a similar experiment proposed that the slowness was the result of a planning of movements that does not take into account the actual capability of the lesioned motor system and that generates a not realistic motor plan. This latter could be a hypothesis for the affected hand but does not explain the results for the unaffected hand.

We found that patients with right-sided strokes did not exhibit temporal congruence while left-sided stroke patients did. For right-sided stroke patients, Malouin et al. (2012) already found a weaker correlation between executed and imagined movements, meaning that these patients could not predict the time necessary to perform the movements or to maintain stable the frequency. They hypothesized that imagining movements was more demanding in right-sided stroke patients due to damage to a fronto-parietal network involved in visuospatial processing. This could be confirmed by the results of another fMRI study in stroke patients (Dettmers et al., 2015). In this study, patients with lesions in the left hemisphere had a higher activation level than those with lesions on the right hemisphere in visual processing (fusiform, lingual gyri, and dorsal premotor regions). They also had more vivid imagery experiences. Furthermore, there is electrophysiological evidence that, in right hemispheric strokes, motor facilitation (corticospinal excitability) during motor imagery does not occur either in the right or the left hemispheres (Stinear et al., 2007) comparing to left hemispheric strokes.

### Limitations

This study is not without its limitations. First, this study is a transversal study and a not longitudinal one. Indeed, it would have been interesting to study the evolution and recovery of implicit and explicit motor imagery performances and determine if they correlate with changes in motor performance. De Vries et al. (2011) suggest that early after stroke, motor imagery ability is more related to visual imagery than actual motor function.

Second, our population size was too small to investigate effects of right-hemisphere neglect. Neglect and anosognosia are known to hinder motor imagery and could lead to a dissociation between visual and kinesthetic abilities (De Vries et al., 2013). This small sample size hampered also the direct comparison of coefficient correlations in the temporal congruence analysis and the impact of side in the explicit task was analyzed on the presence or not of a significant *p*-value.

Third, including another RT task (for example motor task) could have been interesting to prove that the slowness of the RTs was specific to the motor imagery. However, a motor RTs task requires also cognitive and executive processes that may be disturbed in the subacute stage of stroke.

Finally, specific brain lesions, such as damages to the parietal lobe (Sirigu et al., 1996) or basal ganglia (McInnes et al., 2015) are known to impair motor imagery abilities. This would have been interesting to consider in this study but our sample size was too small to disentangle the effect of side and location together.

Despite these limitations, our study has several advantages. We performed both implicit and explicit motor imagery tasks in acute patients with motor deficits representative of a typical clinical population.

## Conclusion

These results suggest that acute stroke patients exhibit evident motor imagery deficits. Moreover, the impact of lesion side is different with respect to the type of motor imagery. Left-sided and right-sided stroke patients exhibited bilateral impairment in solving implicit motor imagery tasks whereas moreover right-sided strokes seemed to yield less correlation between executed and imagined hand movements in the explicit imagery task. Taking into account the discrepancies induced by the side of the lesion, using mental practice for upper limb rehabilitation should be used with caution, especially in right-sided strokes that exhibited both impairment in implicit and explicit imagery performances. Whatever, the more our understanding of motor imageries abilities evolves, the better the real potential of mental practice as a tool of upper limb rehabilitation can be determined (Kraft et al., 2015).

## Author Contributions

CK, EM, YS, and CR have met these 4 criteria: Substantial contributions to the conception or design of the work; or the acquisition, analysis, or interpretation of data for the work. Drafting the work or revising it critically for important intellectual content. Final approval of the version to be published. Agreement to be accountable for all aspects of the work in ensuring that questions related to the accuracy or integrity of any part of the work are appropriately investigated and resolved.

## Conflict of Interest Statement

The authors declare that the research was conducted in the absence of any commercial or financial relationships that could be construed as a potential conflict of interest. The reviewer AS and handling Editor declared their shared affiliation, and the handling Editor states that the process nevertheless met the standards of a fair and objective review.

## Footnotes

1 http://www.mie-uk.com/pgripmyo/index.html
